# Sexually Dimorphic Effects of Aromatase on Neurobehavioral Responses

**DOI:** 10.3389/fnmol.2018.00374

**Published:** 2018-10-15

**Authors:** Dusti A. Shay, Victoria J. Vieira-Potter, Cheryl S. Rosenfeld

**Affiliations:** ^1^Nutrition and Exercise Physiology, University of Missouri Columbia, MO, United States; ^2^Bond Life Sciences Center, University of Missouri Columbia, MO, United States; ^3^Thompson Center for Autism and Neurobehavioral Disorders, University of Missouri Columbia, MO, United States; ^4^Department of Biomedical Sciences, University of Missouri Columbia, MO, United States

**Keywords:** estrogen, brain, testosterone, ArKO, locomotor activity, breast cancer, Alzheimer’s disease, memory

## Abstract

Aromatase is the enzyme responsible for converting testosterone to estradiol. In mammals, aromatase is expressed in the testes, ovaries, brain, and other tissues. While estrogen is traditionally associated with reproduction and sexual behavior in females, our current understanding broadens this perspective to include such biological functions as metabolism and cognition. It is now well-recognized that aromatase plays a vital lifetime role in brain development and neurobehavioral function in both sexes. Thus, ongoing investigations seek to highlight potentially vital sex differences in the role of aromatase, particularly regarding its centrally mediated effects. To characterize the role of aromatase in mediating such functions, effects of aromatase inhibitor (AI) treatments on humans and animal models have been determined. Aromatase knockout (ArKO) mice that systemically lack the enzyme have also been employed. Humans possessing mutations in the gene encoding aromatase, *CYP19*, have also provided critical insight into how aromatase affects brain function in a possible sex-dependent manner. A better understanding of how AIs, used to treat breast cancer and other clinical conditions, may detrimentally affect neurobehavioral responses will likely promote development of future therapies to combat these effects. Herein, we will provide a critical review of the current knowledge of sex differences in aromatase regulation of various neurobehavioral functions. Although many species have been used to better understand the functions of aromatase, this review focuses on rodent models and humans. Critical gaps in our present understanding of this area will be considered, and important future research directions will be discussed.

## Introduction

Aromatase (encoded by the *Cyp19*/*CYP19* gene) is the rate-limiting enzyme responsible for the unidirectional conversion of androgens to estrogen (E2) in gonadal and extra-gonadal tissues (Blakemore and Naftolin, 2016). It is required for E2 synthesis in males and females in steroidogenic tissues/organs, such as gonad, adipose tissue, bone, and brain (Nelson and Bulun, 2001). In mammalian females, aromatase activity in extra-ovarian tissues provides the sole source of E2post-natural or surgical menopause (or ovariectomy in animal models). Yet, aromatase is essential throughout the lifespan in males and females.

Naftolin et al. (1971, 1972) discovered aromatase activity in the hypothalamus of the human fetus and went on to establish aromatase activity in the rat brain as well. These findings led to the “aromatization hypothesis,” which proposes that developmental expression of aromatase in certain brain regions at critical time windows is required for permanent masculinization (Naftolin et al., 1972; Wright et al., 2010). This hypothesis challenged the previously accepted “organizational-activational hypothesis,” postulating that sexual differentiation is driven primarily by the presence or absence of testosterone (T) in the fetal brain in response to XY or XX chromosomal status, respectively (Wright et al., 2010). However, we now recognize the synergistic relationship between androgens and E2 to orchestrate masculinization and feminization of the brain, as well as the potential for genetic (e.g., absence of a Y chromosome) and epigenetic influences (e.g., DNA methylation and/or chromatin modifications) to affect these physiological responses in several brain regions, including the hypothalamus, amygdala, and pre-optic area (Konkle and McCarthy, 2011; Matsuda et al., 2011; McCarthy et al., 2017; Rosenfeld, 2017).

In order to disentangle the complex interactions driving neurobehavioral responses to aromatase activity, research to date has utilized genetic (i.e., manipulation of the aromatase gene, *Cyp19*) and pharmacologic [i.e., aromatase inhibitor (AI) therapy] means. However, species comparisons of aromatase function pose problems due to sometimes stark inter-species differences. For example, rodent species exhibit short gestational period and tend to be polygynous, whereas, humans have long pregnancy duration and most societies are monogamous and biparental; this makes investigation of the precise role of aromatase during gestation difficult. Even so, early post-natal brain development in rodents resembles the critical window for human fetal brain development that occurs during the third trimester (Rice and Barone, 2000; Selevan et al., 2000; Howdeshell, 2002) making the rodent model appropriate for investigation of how aromatase affects brain development specifically. Thus, this review will focus on sex differences in aromatase expression and activity in the brain of mammalian species only. However, it is important to note that non-mammalian species are also known to express aromatase in the brain, and research in non-mammalian species (e.g., birds and fish) has also proven useful; the reader is referred to these cited references for further analyses on those species (Balthazart, 1997; Cao et al., 2012; Vahaba and Remage-Healey, 2018).

Aromatase-specific whole-body knock-out (ArKO) mice (bred on the C57Bl6 background) have been developed that constitutively lack aromatase activity throughout all stages of development. This has been a useful animal model to discern the role of aromatase in physiology and behavior of both sexes (Boon et al., 2010; Borbelyova et al., 2017). There are also 15 known cases of aromatase deficiency in humans (8 men, 7 women) (Boon et al., 2010). In women, aromatase deficiency is typically diagnosed early in life due to pseudo-hermaphroditism at birth and the absence of pubertal menstruation in females, with E2 supplementation generally being curative (Boon et al., 2010). In men, aromatase deficiency tends to be diagnosed later in life with pubertal development being typically normal, although infertility tends to progress with maturity (Boon et al., 2010).

Pharmacologically, AI (Anastrozole, Letrozole) can be administered to down-regulate aromatase activity. AI are primarily used as adjuvant hormone therapy in pre- and post-menopausal women with breast cancer and in less frequent cases of male metastatic breast cancer but are also employed in the treatment of other hormonal conditions (Phillips et al., 2011; Bulun et al., 2012). In boys and girls, AI can assist in the treatment of a number of sex hormone-related conditions in which E2 is over-expressed (Wit et al., 2011). In adolescent males, AI can be used to correct short stature due to pubertal delay (Wit et al., 2011). In sexually mature men, AI can be used to assist with low sperm count and motility, which often contributes to infertility (Schlegel, 2012; Borbelyova et al., 2017). In later adulthood, males often experience a slow, but steady decline in serum T, thus increasing their ratio of E2 to T, which may result in enlarged or painful breasts in men (Tan et al., 2014). This can be treated with an AI to reduce the amount of androgens being converted to E2 and ultimately increase serum T concentrations that naturally decline with age (Tan et al., 2014; Borbelyova et al., 2017).

## Sex Differences in the Distribution and Regulation of Aromatase

**FIGURE 1:**
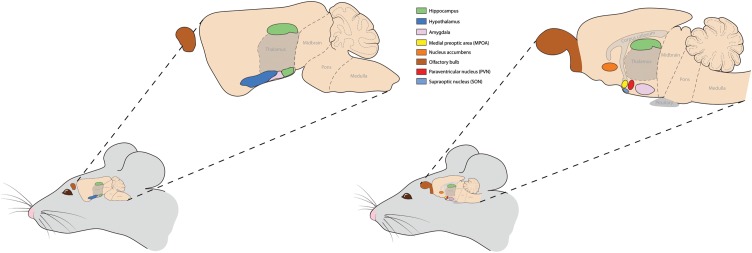
Predominant brain regions expressing aromatase or the *Cyp19* gene in mice and likely other animals. Two sagittal brain sections are illustrated to show the various brain regions, including the olfactory bulbs, hippocampus, hypothalamus, amygdala, and nucleus accumbens. Within the hypothalamus, the medial preoptic area (MPOA), paraventricular nucleus, and suproptic nucleus express high amounts of this enzyme. These brain regions are highlighted as aromatase expression in them guides many of the behaviors discussed herein and in many cases do so in a sex-dependent manner.

*CYP19/Cyp19* is widely expressed in the central nervous system (CNS) of both sexes, as well as in different neuronal cell types, with levels of mRNA expression being greatest in regions associated with sexual differentiation and corresponding closely with expression of E2 receptor alpha (ESR1) and beta (ESR2), as well as androgen receptors (AR) (Martinez-Cerdeno et al., 2006; Roselli et al., 2009; Bowers et al., 2010; Wright et al., 2010; Stanic et al., 2014; Tabatadze et al., 2014; Fester et al., 2017; Lorsch et al., 2018). Predominant brain regions in male and female species expressing *Cyp19* are illustrated in **Figure 1**. The presence of aromatase in various neural cell types suggests that the enzyme functions in an autocrine and paracrine fashion, and in some instances, independently of circulating sex hormone levels and presumably contribute to sex differences in the development of neurons during gestation and following injury and will be discussed in later sections.

Sex differences in *Cyp19* expression in rodent brains and varies by region. Tabatadze et al. (2014) found that in comparing intact and gonadectomized male and female Sprague-Dawley rats, intact males had the highest levels of *Cyp19* gene expression in the amygdala, bed nucleus of stria terminalis (BNST), and pre-optic area, while no sex differences were observed in the dorsal hippocampus and cingulate cortex (Tabatadze et al., 2014). Similarly, Stanic et al. (2014) determined that female mice have lower aromatase protein expression in BNST and medial amygdala, when compared to males (Lorsch et al., 2018). Furthermore, steroidal control of *Cyp19/CYP19* expression may be sex-specific. Adult, male Sprague-Dawley rats (∼60 days old) have higher aromatase activity in specific micro-dissected sub-regions of the hypothalamus and amygdala compared to females, while other subsections displayed no sex differences (Roselli et al., 1985). After castration, brain regions with high or moderate aromatase activity decreased to levels comparable to females, and were restored after treatment with T (Roselli et al., 1985).

Similar to the animal species described above, the adult human brain exhibits aromatase expression in several regions, in particular the hippocampus, amygdala, hypothalamus, frontal cortex, and temporal lobe, whereas no sex differences have been identified to date (Sasano et al., 1998; Stoffel-Wagner et al., 1999; Yague et al., 2006). However, PET studies with [*N*-methyl-^11^C]vorozole have revealed that humans uniquely possess high levels of aromatase in the thalamus and medulla (inferior olive), but that study did not investigate sex differences in aromatase expression in those two brain regions (Biegon et al., 2010). In human pituitary tissue, there was a non-significant trend for males to show elevated levels of aromatase expression compared to females, but limited sample tissue prevented definitive conclusions from being drawn (Kadioglu et al., 2008). Caution should be taken in interpreting those findings due to the difficulty in performing experiments in human subjects and the limited availability of human brain tissue; however, it is notable that abundant human data demonstrate sex differences in cognitive functions, such as learning and memory, addiction, and motivation, as well as in various pathologies such as epilepsy, Alzheimer’s, schizophrenia, anxiety and depression (Beyenburg et al., 1999; Cherrier et al., 2001, 2005; Butler et al., 2010; Blakemore and Naftolin, 2016; Op de Macks et al., 2016; Alarcon et al., 2017). Thus, aromatase likely plays a vital role in modulating these sexually dimorphic differences.

The regulation of *CYP19/Cyp19* expression and aromatase activity in the brain involves genomic, non-genomic, and epigenetic factors. This regulation can vary throughout the lifespan, as well as between sexes, (Boon et al., 2010; McCarthy et al., 2017) and might play an important role (pre and postnatally) in sexual programming of the brain, along with hormone status and even neuroimmune mediation (e.g., reactive oxygen species (ROS), cytokines, and prostanoids) (Konkle and McCarthy, 2011; McCarthy et al., 2017).

There is mounting evidence that aromatase is also rapidly regulated via non-genomic mechanisms involving direct phosphorylation of the aromatase enzyme under conditions that promote protein phosphorylation, in particular high levels of calcium, magnesium, and ATP (Balthazart et al., 2003; Fester et al., 2017). These findings suggest that non-genomic regulation of aromatase can rapidly increase local E2 levels within the brain, thereby allowing for quick neurobehavioral responses to fluctuating environmental conditions (Balthazart et al., 2003; Fester et al., 2017). Interestingly, a protein heavily involved in brain synaptic plasticity, synaptopodin, is directly regulated by E2 and is expressed more strongly in female vs. male hippocampus (Fester et al., 2017). Non-genomic regulation of aromatase is responsible for the effect on synaptopodin, since an increase in intracellular calcium release inhibited aromatase activity in the hippocampus and downregulated synaptopodin expression in males and females, as discussed in more detail in a later section (Fester et al., 2017).

## Sex Differences in Aromatase During Critical Periods of Development

Evidence suggests that there are two aromatase systems that exist within the adult mammalian brain: a gonad-sensitive hypothalamic system, and a non-gonad-sensitive limbic system (Naftolin, 1994). Brain regions responsible for sexual differentiation and behavior, such as the hypothalamus and medial preoptic area, are dependent on gonadal function and sex-steroid secretion, supporting the aromatization hypothesis. In other brain regions, such as the cerebral cortex, amygdala, and hippocampus, aromatase activity is present but is gonad-function independent. These areas in adult rats also do not respond to removal of T or E2 under experimental conditions (Abdelgadir et al., 1994). In 2–3 year old rams (*Ovis aries)*, castration decreases aromatase expression in the hypothalamus but not in the amygdala (Roselli et al., 1998).

A critical embryonic window exists during which the brain is programmed to be either masculinized or feminized (McCarthy et al., 2017). This critical period in rats is predominantly during the last 5–7 days of gestation with rodent gestation lasting approximately 21 days but recent evidence suggests that this critical period can extend into the post-natal period (Wright et al., 2010; Nugent et al., 2015). During the critical period of short-gestation mammals (e.g., rodents), males are exposed to higher levels of E2 than females, as demonstrated by greater concentrations of T coupled with an increase in aromatase activity (Lephart, 1996). After the critical period, the brain cannot be reprogrammed with endogenous or exogenous T or E2, although it may still be vulnerable to epigenetic influences (McCarthy et al., 2017). This study revealed that pharmacological inhibition of DNA methyltransferase mimics the action of E2 in masculinizing the brain, even if administration occurs after the so-called critical period (Nugent et al., 2015).

During the development of long-gestation mammals (e.g., primates, sheep, and guinea pigs), aromatase may also affect brain regions not typically associated with sexual differentiation, such as the hippocampus, cortex, and amygdala. These areas also must be programmed at critical windows of time. For instance, in sheep which typically have a 147–150 day gestation period, the brain is sculpted between 60 and 90 gestational days; whereas in non-human primates with a 165 gestational period, this occurs at 40–60 gestational days (Roselli and Resko, 1986; Roselli et al., 2014). These effects are not always androgen-dependent, and both gene expression and enzyme activity may fluctuate throughout the lifespan within individual brain regions and across species (Roselli and Resko, 1986; Connolly et al., 1994; Roselli et al., 2003). Male and female rhesus macaques (*Macaca mulatta*) were gonadectomized at 98–103 days gestation and were surgically removed from the uterus at 120 days of gestation (Roselli and Resko, 1986). In the cerebral cortex and amygdala aromatase activity was significantly greater in males than females, but similar sex differences were not observed in the preoptic area or hypothalamus (Roselli and Resko, 1986). Conversely, analysis of fetal sheep brain (at gestational days 53, 65, 85, 100, 120, and 135) by Roselli and Stormshak (2012) revealed that *Cyp19* expression levels in the amygdala was similar across days tested in males and females (Roselli and Stormshak, 2012). This study also revealed that both sexes demonstrated a similar temporal pattern of *Cyp19* expression in the preoptic area, suggesting that in both non-human primates and sheep aromatase expression in this brain region is not influenced by sexually dimorphic factors.

It is important to also recognize that steroid hormones exert an activational role at the time of sexual maturity that varies across animal species (Bell, 2018). Studies of sex differences in central aromatase during puberty are extremely limited, especially in mammals. One study gonadectomized male and female Sprague-Dawley rats at post-natal day (PND) 30 (considered prepubertal), and by PND56 (post-pubertal), identified significantly reduced aromatase activity in the BNST, medial preoptic nucleus, and preoptic area, but not in the anterior hypothalamus or medial amygdala in both sexes (Roselli and Klosterman, 1998). Relative sex differences in aromatase activity in these brain regions were observed, as males showed higher basal levels at the outset. In contrast, a similar study in intact mice found no sex differences in *Cyp19* mRNA expression in the BNST and hypothalamus during the peripubertal phase (PND 20–60) (Kanaya et al., 2018). In sheep, prenatal exposure to 1,4,6-androstatriene-3,17-dione (ATD) did not affect the pubertal development of females, but caused a significant decrease in mounting behavior of males at 18 months of age without inducing feminization of males (e.g., receptive behavior and luteinizing hormone LH surge mechanism) (Roselli et al., 2006). Taken together, the limited available evidence suggests that central aromatase deficiency may affect males more than females, at least regarding sexual behaviors.

## Aromatase and Sociosexual Behaviors

Sociosexual behaviors such as lordosis, mounting, and preference for non-volatile pheromones (and potentially volatile odors from urine/bedding) assists in mate selection and reproduction in several mammalian species. These behaviors may be regulated by aromatase. In rodents, olfaction detection and perception are considered socio-cognitive behaviors in that odor recognition of same and different sex individuals guides subsequent socio-behavioral responses, i.e., fighting or mating behaviors. Two olfactory systems exist in rodents and likely mediate diverging purposes (Bakker, 2003). The main olfactory system is primarily associated with detection of volatile odors and is traditionally associated with finding food and detection of predators (Bakker, 2003). The accessory olfactory system detects non-volatile pheromones and assists in mate selection and reproductive behaviors and is known to be sexually dimorphic (Bakker, 2003).

When examined separately, Bakker (2003) found genotype (ArKO vs. WT), but no sex differences in the role of aromatase in the development of the main olfactory pathway, and a lack of an effect on the accessory olfactory pathway (Bakker, 2003). The findings suggest that aromatase is important for development of the main olfactory system in both sexes, but not the accessory olfactory system (Bakker, 2003).

In a separate but supporting study using the same mouse model, Bakker et al. (2010) applied urinary odors directly to the nasal region of all mice tested in order to activate both the main and accessory olfactory systems. Here, the investigators detected both genotype and sex differences. In response to urinary odors of opposite sex conspecifics, wild-type (WT) females exhibited a significant increase in hypothalamic c-fos expression of kisspeptin neurons (an upstream regulator of gonadotropin releasing hormone- GnRH secretion), while such responses were blunted in ArKO females (Bakker et al., 2010). In WT males, minimal increase in c-fos expression was noted, while no increase was evident in ArKO males in response to the odor cues of estrus females (Bakker, 2003; Bakker et al., 2010). Taken together, a sex difference may exist in the role of aromatase in mate selection, possibly via influence on kisspeptin neurons, regulated secondarily the GnRH/LH system. Interestingly, expression of kisspeptin mRNA (*Kiss1*) and kisspeptin neuronal activity were reduced in both ArKO males and females, seemingly contradicting the previous findings by Bakker et al. (2010) that suggests kisspeptin neurons are essential for regulating GnRH in the hypothalamus and indirectly, the LH surge. The conflicting findings suggest aromatase plays a role in this pathway but is not the primary enzyme involved. (Szymanski and Bakker, 2012).

However, further examination of the GnRH/LH system by Szymanski and Bakker (2012) determined that administration of E2 + progesterone to WT and ArKO females and ArKO males (between 3 and 6 months of age) successfully produced an LH surge (considered a female pattern of secretion). However, in WT males, defeminization occurred normally, thus rendering them unable to produce a later LH surge in response to these ovary-associated hormones (Szymanski and Bakker, 2012).

Pharmacological manipulation of aromatase has also been utilized to examine the effects of the removal of E2 during gestation on later adult sexual behaviors. Olvera-Hernandez et al. (2015) reported a feminized-pattern of results in male Wistar rats after prenatal exposure to Letrozole (0.56 μg/kg of the dam) with 30% of males showing same-sex preference and 33% displaying lordosis. However, 44% of treated males also displayed a completely masculinized pattern of sexual behavior, i.e., mounting behaviors, intromission, and ejaculation toward receptive females (Olvera-Hernandez et al., 2015), suggestive that some males might be resistant to the effects of this AI. The mechanism by which prenatal exposure to AI affects adult sexual behavior could be epigenetic in origin (Matsuda et al., 2011; Nugent et al., 2015; McCarthy et al., 2017).

In support of an epigenetic component and E2 signaling on adult socio-sexual behaviors, El Hajj Chehadeh et al. (2014) observed decreased *Cyp19* expression in the olfactory bulbs of 21-day old female rat pups whose mothers were fed a methyl-donor deficient diet throughout gestation (El Hajj Chehadeh et al., 2014). In addition, these same pups also exhibited impaired olfactory discrimination during the lactation and weaning periods (El Hajj Chehadeh et al., 2014). While olfactory cues in the diet primarily triggers the main olfactory system in rodents, pheromones are also likely implicated in pup interaction with dams (as with mate selection later in life) and thus, further investigation is necessary to elucidate the effects of DNA methylation and E2/E2 receptor signaling on the accessory olfactory system and sexual behaviors later in life.

Other rodent models have been used to understand how systemic and central aromatase activity regulates other socio-sexual behaviors, in particular prairie voles (*Microtus ochrogaster*) that are monogamous and demonstrate biparental care. Conflicting parenting results have been obtained in prairie voles treated with AI, which are likely due to when the animals were exposed to the pharmaceutical agent. Female virgin prairie voles normally exhibit infanticidal behavior when exposed to pups, while virgin males exhibit normal parental behaviors (Lonstein and De Vries, 2000). In this study, prenatal exposure to AIs (via subcutaneous injection of 1 mg ATD on gestational days 15–19) or post-natal exposure to the same AI (0.5 mg ATD on PND1-7) did not alter the parental or infanticidal behaviors of the prairie voles in adulthood (Lonstein and De Vries, 2000). However, female offspring prenatally exposed to ATD had higher levels of parental care compared to T propionate-treated females. On the other hand, treatment of male prairie voles with ATD (subcutaneous injection of 0.5 mg for PND8-14) or flutamide (0.5 mg for PND8-14) reduced alloparental behavior in 21-day old males with ATD-treated males demonstrating an increase in sniffing behavior but also an increase in latency to lick and huddle the pups, whereas flutamide-treated males had increased latency to enter the pup cage and probability of retreat from initial contact (Kramer et al., 2009).

To test effects of developmental exposure to AI on later reproductive behaviors in prairie voles, male and female pups were treated daily with ATD (injection 0.5 mg on PND1-7) or vehicle control (Northcutt and Lonstein, 2008). ATD-treated females were the recipients of fewer mounts and thrusts, suggestive of reduced attractiveness to males. Males treated neonatally with ATD showed a demasculinized pattern in tyrosine hydroxylases expression in the anteroventral periventricular preoptic area and ESR1 expression in the medial preoptic area. Female guinea pigs (*Cavia porcellus*) subjected to prenatal ATD exposure had decreased mounting activity but comparable responses were not observed in males subjected to similar AI exposure (Roy, 1992). However, another study testing the effects of developmental exposure to ATD in guinea pigs (gestational day 30 to day 55) reported that both males and females were irresponsive to the negative feedback effects of E2 benzoate or LH secretion, and ATD-treated males demonstrated increased lordosis behavior compared to control males (Choate and Resko, 1994). However, no changes in mounting or lordosis behavior were evident in ATD-treated female guinea pigs (Choate and Resko, 1994).

Taken together, the above data provide mixed results as to the role of an early prenatal aromatase surge in guiding later sociosexual behaviors. The results are muddled by the fact that different AI and doses have been tested, variation in developmental exposure periods, and specific tests used to measure sociosexual behaviors. Moreover, the studies have used different animal models that demonstrate divergent reproductive strategies (polygynous vs. monogamous) that assuredly will influence the outcome. The ideal experiment should test the same AI dose at the same gestational period in rodent models displaying these two types of mating systems.

## Sex-Specific Role of Aromatase in Stress-, Anxiety-, and Depressive-Like Behaviors

Anxiety and depression are two of the most widely diagnosed mental illnesses today with women being more likely to be diagnosed than men (Altemus et al., 2014). While animals do not experience stress or depressive behaviors per se, behavioral tests, such as the elevated plus maze EPM, tail suspension test, and forced swim test (FST), can be used to measure behaviors that are suggestive of anxiety- and depressive-like behaviors. In the EPM, decreased entries into the open arms might suggest anxiety-like behaviors, and increased time spent immobile in the tail suspension test and FST are indicative of depressive-like behaviors in that the animal is giving up trying to escape an aversive (water or suspension) stimulus. These emotive behaviors are considered amygdala-dependent (Gray, 1999) and are collectively considered in this section. With the usage of such rodent tests, there is still limited data comparing aromatase regulation of male and female responses in these categories. Most current studies have examined males or females only, making sex comparisons across reports difficult due to non-uniform procedures to measure these behaviors, such as type of test, genetic or pharmacological manipulation, AI administered, time and duration of AI administration, or even species/rodent strain.

Kokras et al. (2018) showed that female Wistar rats (3 months old) enter the center of an open field test (OFT), indicative of lower anxiety levels, more often than males, but this affect was eliminated by ovariectomy or Letrozole treatment (intraperitoneal injection of 1 mg/kg letrozole for 8 days) indicating that systemic E2, and not central aromatase activity, may be responsible for the primary anxiolytic effect observed in females (Kokras et al., 2018). In a separate study, Letrozole (1 mg/kg subcutaneously for 14 days) induced an anxiogenic effect in intact male Lewis rats which spent, on average, 95% less time in the open arms of the EPM when compared to control males, while intact females given the same AI did not differ significantly from controls, suggesting AI-induced anxiety-like behaviors in males but not females (Borbelyova et al., 2017).

Using the ArKO mouse model, Dalla et al. (2004, 2005), demonstrated that genotype (ArKO vs. WT) did not affect male performance, but female ArKO mice displayed more passive behaviors (e.g., decreased mobility) during the FST, an inherently stressful behavioral test for terrestrial animals that is designed to measure depressive-like behaviors, indicative of elevated depressive-like behaviors (Dalla et al., 2004, 2005). However, Kokras et al. (2018) found that Letrozole did not affect behavior of either sex in this test, although aromatase activity was decreased in the hypothalamus of male rats after behavioral testing (Kokras et al., 2018). This study demonstrated that genotype did not affect male performance, but female ArKO mice displayed more passive behaviors (e.g., decreased mobility) during the FST, indicative of elevated depressive-like behaviors (Dalla et al., 2004, 2005). In contrast, male but not female ArKO mice showed an age-related reduction in the prepulse inhibition of the acoustic startle test, which measures ability of rodents to habituate to an external stimulus (van den Buuse et al., 2003). Collectively, these findings suggest that central E2 synthesis may differentially affect specific aspects of mental health with a potentially greater role in female rover male rodents.

In contrast to the above findings, a study with Sprague-Dawley rats found that acute stress decreased circulating E2 and T in females, while these hormones remained unchanged or in some cases increased, whereas no differences in hypothalamic *Cyp19* mRNA expression were detected (Lu et al., 2015). There is increasing recognition that stressful events during pre- and/or post-natal development may contribute to future development of anxiety or depression in adulthood; whether sex plays a role in the relationship between stress and development is not fully known and is an important area for future work. Such sex-specific developmental programming [known as the developmental origin of adult health and disease (DOHaD) concept (Weinstock, 2007; Wadhwa et al., 2009)] may involve aromatase activity. In support of this notion, maternal stressors, such as prolonged restraint, suppresses aromatase activity in the hypothalamus and amygdala of the male rat (unspecified strain) offspring, causing developmental changes in these brain regions potentially contributing to future anxiety, depression, or learning deficits (Weinstock, 2007). Only a few studies have examined the role of aromatase in the fetal brain as a potential mediator of prenatal programming effects. One of those studies tested how exposure of female Wistar dams to ATD during late gestation would affect later offspring anxiety and emotional behaviors when tested at 1 month of age in the OFT and EPM and found that maternal exposure to ATD increased anxiety-like behaviors in both sexes (Ordyan et al., 2007). With the increased incidence of anxiety and depression in adults, as well as attention deficit disorder in children, further research in this area is vital to understanding the effects of this increase in diagnoses and possible involvement of aromatase in offspring and even descendants through transgenerational inheritance.

## Sexually Dimorphic Effects of Aromatase: Possible Role in Alzheimer’s Disease?

The presence of aromatase in mammalian hippocampus suggests that E2 plays a role in learning and memory (Overk et al., 2012; Vierk et al., 2012; El Hajj Chehadeh et al., 2014). Deficits in learning and memory are also a hallmark of Alzheimer’s disease (AD) and sex differences have been observed with women overall being at an increased risk (Medway et al., 2014). The Epistasis Project by Medway et al. (2014) determined that 3 of the 4 single-nucleotide polymorphisms (SNPs) of *CYP19* tested were associated with increased risk for AD in women but not in men (Medway et al., 2014). Another human cohort study, OPTIMA, examined nine polymorphisms in the *CYP19* gene in 207 cases of AD, 23 cases of mild cognitive impairments (MCI), and 233 control men and women. Consistent findings between those polymorphisms and both AD and MCI cases were observed, and significant interactions between select polymorphisms and sex were also noted. Remarkably, all associations between *CYP19* polymorphisms and AD were identified exclusively in women (Butler et al., 2010). Three SNPs (res12907866, rs17601241, and rs4646) in *CYP19* were assessed in 319 AD patients and 110 controls representing men and women, and the investigators determined that while none of these CYP19 SNPs increased the risk for AD, women with the rs4636 genotype carrying a T allele were more likely to have an earlier-onset of AD (Corbo et al., 2009).

While aromatase suppression has been associated with increased risk for AD as summarized above, meta-analyses have been inconclusive about potential sex differences (Roselli et al., 1998; Medway et al., 2014; Prange-Kiel et al., 2016). Rodent studies have been somewhat useful in shedding light on the effects of aromatase on risk for AD-like signs, especially in females. Sex differences in intact 5xFAD mice (a mouse model for AD) were observed such that the hippocampi of female (but not male) mice had significantly lower *Cyp19* expression and protein activity when compared to WT counterparts (Prange-Kiel et al., 2016). Sex differences were also observed with Anastrozole administration (0.4 mg/animal/day via solid hydration gel matrix) to intact 9-month-old female 3xTgAD mice (another mouse model of AD), which significantly increased Aβ_1-40/42_-immunoreactivity (a marker of AD pathology) in the hippocampus of intact AI-treated mice (Overk et al., 2012). No significant differences were seen between gonadectomized males and females with or without anastrozole-treatment, suggesting that these effects are due to systemic E2 rather than local aromatase activity (Overk et al., 2012).

In another study, aromatase inhibition completely abolished long-term potentiation (LTP); an electrophysiological parameter of memory, in the hippocampus of 12-week old female C57Bl/6 mice, while treated males of the same age only experienced a 20% reduction in LTP after 7 days (Vierk et al., 2012). Surprisingly, ovariectomy reduced this effect in females with Letrozole producing only a 50% decrease in LTP after 7 days of treatment, suggesting that central aromatase activity and gonadal estrogen work synergistically to modify LTP in the hippocampus of females (Vierk et al., 2012). In neonatal (PND0) Sprague-Dawley rats, exogenous E2 has been shown to promote cell proliferation and survival in hippocampi of females but not males, and this effect persists through PND21 (Bowers et al., 2010). Surprisingly, subcutaneous injection of the AI formestane (100μg/0.1 mL in sesame oil at PND0) significantly decreased hippocampal cell proliferation in male Sprague-Dawley rats but had no effect on females (Bowers et al., 2010).

To further examine potential interactions between aromatase suppression and AD, APP23-ArKO mice have been generated in order to assess the interactions between an increased risk for AD (as in the APP23 mouse model) and a systemic absence of E2 (as occurs in the ArKO model) (McAllister et al., 2010; Li et al., 2013). App/Cyp19 heterozygous ovariectomized females have reduced Aβ plaques and reduced expression of β-secretase 1 (BACE1, enzyme responsible for cleavage of amyloid precursor protein- APP) mRNA and protein expression following E2 or genistein treatment (Li et al., 2013). On the other hand, APP/ArKO males show reduced brain plague formation, enhanced cognitive functions, increased neprilysin, involved in Aβ clearance), and reduced BACE (McAllister et al., 2010). Collective findings in this male line suggests that elevated T due to absence of CYP19 protects against AD-like signs (McAllister et al., 2010). Elevated T might confer protection against this disease in males by suppressing BACE1 activity resulting in reduced β-amyloid production and increased neprilysin to stimulate degradation of any existing β-amyloid within the brain. Overall, the results reveal that the mechanisms by which aromatase, and thus sex hormones, affect cognition and risk for AD are complex and may involve aromatase mediated differences and/or changes in spine synapse density, *CYP19* gene expression, and aromatase activity (Fester et al., 2017).

## Sex-Specific Regulation of Locomotor Activity by Aromatase

Possibly the most limited area of research on sex differences in aromatase is in its effects on physical/locomotor activity. It is known from many rodent studies that intact females are significantly more active than age-matched males (Hong et al., 2009) and preclinical studies in rodents have implicated central E2 signaling in mediating increased physical activity in females (Xu et al., 2015). Moreover, studies in rodents and women provide evidence that aromatase activity may directly affect physical activity, and this effect of aromatase may be sex-specific. Not surprisingly, the ArKO female mouse model demonstrates compromised physical activity (Jones et al., 2001). Notably, this phenotype of ArKO females is even more pronounced than similar observations in ovariectomized WT females, who lack gonadal E2 but presumably have normal aromatase enzyme activity and E2 synthesis in the brain (Jones et al., 2000). It is uncertain whether ArKO males are also significantly less active than WT controls, as only females have been tested to date. There is, however, limited evidence involving pharmacological AI in male mice. In one study, administration of AI (SC 500μl bolus with 250 mg/kg exemestane + injection of Letrozole at 0.5 μg/μl for 7 days) had no effect on voluntary wheel running, while orchidectomy reduced wheel running (Bowen et al., 2011). These findings imply that systemic androgens/T, and not E2, affects wheel running in male mice. While ArKO mice of both sexes have been shown to be metabolically dysfunctional (Jones et al., 2000, 2001), the evidence suggests that aromatase activity offers some metabolic protection in both sexes, but affects physical activity behavior only in females.

Few studies have examined the mechanisms by which aromatase affects physical activity directly, so much of the available evidence includes secondary measurements of locomotor activity as assessed in association with another major outcome (i.e., anxiety-like, depressive-like, or schizophrenia-like behaviors). One experiment conducted in an attempt to understand the role of E2 in schizophrenia discovered that female and male ArKO mice experienced reduced psychotropic drug-induced hyperactivity in an OFT after administration of apomorphine, MK-801, and amphetamine while WT counterparts were unaffected, although this effect was not as pronounced in male ArKO mice (Chavez et al., 2009). Thus, these results lend support for the idea that males and females are potentially differentially affected by aromatase deletion and consequent locomotor activity. This sexually dimorphic difference is possibly explained by an increased density of dopamine transporters in the caudate putamen of male but not female ArKO mice when compared to WT mice, potentially allowing for a compensatory dopaminergic upregulation in male (but not female) ArKO mice (Chavez et al., 2009).

In contrast, a separate study showed that aromatase inhibition administered via subcutaneous injection had no effect on locomotor activity of neither male nor female middle-aged rats (15 months) when tested in the EPM, which measures both anxiety-like and exploratory behavior (Borbelyova et al., 2017). Similarly, Kokras et al. (2018), who studied younger (i.e., 3 month old) Wistar rats, also reported no sex differences in locomotor activity when measured during an OFT (Kokras et al., 2018). Other reports confirm that locomotor activity is not affected by AI in either sex when it is assessed in an OFT or EPM (Dalla et al., 2004, 2005). However, it is important to consider that all of those above studies only assessed locomotor activity as it relates to other behavioral readouts (i.e., during EPM and OFT assessments of anxiety and/or depressive behaviors) which represents a major confounding variable when assessing spontaneous or motivated physical activity. The role of aromatase activity *per se* on sex differences in physical activity requires more investigation. Better understanding the sex-specific effects of aromatase on physical activity (e.g., potentially decreased motivation to exercise in one or both sexes) may lend insight into the mechanisms driving physical activity, as well as potential side effects of aromatase inhibition in humans. Our current studies seek to examine for potential sex-differences in motivated physical activity using an ArKO mouse model.

## Neuroprotective Role of Aromatase Following Brain Injury

Sex differences in recovery following brain injury and ischemia have been reported (Melcangi et al., 2016). Rodent studies have often concluded that females experience better outcomes in recovery than males (Chavez et al., 2009; Bowen et al., 2011). While the vast majority of work has investigated the neuroprotective effects of E2 (Zhang et al., 2014; Pietranera et al., 2015) the fact that aromatase is expressed in the brain of males and females, and thus contributes to the production of neuroestrogen, potentially implicates a neuroprotective role of aromatase in both sexes.

How aromatase may contribute to recovery from traumatic brain injury or stroke may depend on the level of aromatase expression in that particular brain region, as well as the cell type (e.g., astrocytes of the human temporal cortex), as discussed earlier. Since astrocytes following brain injury or ischemia become reactive, aromatase activity by these cells in particular may mediate neuronal recovery from injury (Garcia-Segura et al., 1999; Melcangi et al., 2016) Indeed, evidence supports the hypothesis that the protective effects of astrocytes is due to E2 availability (i.e., dependent upon aromatase activity), although whether sex differences exist remains unclear (Garcia-Segura et al., 1999).

Lavaque et al. (2006) examined *Cyp19*, P450scc (an enzyme involved in the conversion of cholesterol to pregnenolone, the first step in steroid hormone production), and StAR (regulates cholesterol transport in the mitochondria) expression in the cerebellum of male and female Wistar albino rats at several days of early development (i.e., PND 1, 5, 10, 15, 20, and 30). In both males and females, peak *Cyp19* expression occurred at PND5, with males exhibiting higher expression than females at all ages. In females, an increase in aromatase at PND10 was sequentially followed by an increase in P450scc and StAR (Lavaque et al., 2006). However, the increases in these three enzymes (i.e., aromatase, P450 scc and StAR) were synchronous in males at PND10 and significantly higher than females. Previous studies in males examined the effects of central E2 on neuronal death after administration of 3-acetylpyridine (3-AP - a toxin that produces degeneration of inferior olivary neurons and the consequent degeneration of climbing fiber afferents to cerebellar Purkinje cells) (Lavaque et al., 2006). They found that inhibition of aromatase increased the neurodegenerative effect of 3-AP, while administration of exogenous E2 decreases the effects of 3AP, again supporting the idea that E2 is neuroprotective and that aromatase activity correlates with this neuroprotection (Lavaque et al., 2006). In that study, an increase in P450scc and StAR mRNA was observed in adult male Wistar rats after injection of 3-AP (dose, 60 mg/kg in saline) in the cerebellum (Lavaque et al., 2006). Taken with the findings from previous studies, the evidence suggests that aromatase, StAR, and P450scc all work in tandem to increase steroidogenesis in response to cellular damage (Lavaque et al., 2006). The fact that aromatase, P450scc, and StAR remain unchanged in female rats throughout the estrous cycle suggests that, at least in the cerebellum, it is local and not gonadal E2 production that provides neuroprotection following brain injury and in neurodegenerative diseases (Lavaque et al., 2006; Garcia-Segura, 2008).

## Conclusion

Aromatase is the key enzyme in the E2 production pathway, and its marked expression in the brain highlights its potential importance in many neurobehavioral functions. While differences in aromatase expression and activity may not translate directly to behavioral differences, animal studies indicate that aromatase expression and activity is necessary for proper neurobehavioral development in both males and females. Later in life, sex differences in aromatase may affect risk for neurological diseases, such as depression and AD, which tend to be greater in females than males (Dalla et al., 2004; Ordyan et al., 2007; Overk et al., 2012; Lu et al., 2015; Alarcon et al., 2017; Kokras et al., 2018). Research continues to elucidate the mechanisms by which aromatase affects neurobehavioral programming and sex differences in cognitive and other neurological disorders.

In addition to sociosexual, emotive, and cognitive behaviors regulated by aromatase, emerging evidence suggests aromatase might also affect motivational physical activity. Ovary-intact female rodents are more active than male counterparts (Martin et al., 2003; Afonso et al., 2013) and E2 is known to affect physical activity (Hong et al., 2009; McAllister et al., 2010; Xu et al., 2015). Not surprising, ArKO mice are known to exhibit suppressed physical activity compared to WT. Whether this translates to humans is not clear, but it is noteworthy that AI drugs have been shown to reduce physical activity in humans, a finding with important relevance to women receiving AI to treat breast cancer and other disorders.

Preclinical studies using ArKO and AI-treated rodent models have unraveled some of the multifaceted mechanisms by which aromatase can induce behavioral sex differences. Gonadectomized animal models have also helped tease apart systemic from central effects of aromatase. However, creation of brain-region specific aromatase knockout mice would allow for even more precise assessments on how this enzyme regulates various brain regions and corresponding behaviors in a sex-dependent manner.

While rodent models have provided insight into the role of aromatase in the brain and sex differences, there are potential drawbacks, including differences in gestational period, which might limit translation to humans. Non-invasive methods to image the human brain will aide in our understanding the role aromatase in various brain regions and sex differences. One rapidly evolving method to image in a non-invasive manner the human brain *in vivo* is near-infrared spectro-imaging (Toronov et al., 2005). Structural brain features are determined by spatial light bundles derived from photons traveling to light source detectors placed on top of the head. Positron emission tomography (PET) and a radiolabeled competitive inhibitor of aromatase can also be used to *in vivo* image brain aromatase (Moraga-Amaro et al., 2018). For instance, administration of [^11^C]vorozole and PET performed in female baboons revealed the highest uptake of this compound was detected in the amygdala followed by the preoptic area and hypothalamus, basal ganglia, and cortical areas (Pareto et al., 2013). As mentioned previously, PET studies with [*N*-methyl-^11^C]vorozole have revealed high levels of aromatase in the thalamus and medulla (inferior olive) of humans, which has not been identified in these brain regions of other species (Biegon et al., 2010). In combination with these imaging modalities, it is also important to measure directly enzyme and steroid concentrations in the various brain regions of rodent models and in humans where possible.

Future research with enhanced transgenic and imaging technologies will continue to improve our understanding of the relationship between aromatase and neurobehavioral functions. Such methodologies will help unveil the role of this enzyme in normal cognitive aging, mental illness, neurodegenerative disease, and even traumatic brain injury, and whether those relationships are sex specific. A better understanding of how aromatase contributes to the etiology of such cognitive diseases may lead to improved treatment strategies in men and women.

## Author Contributions

DS, VV-P, and CR researched the area, wrote the original manuscript, and reviewed the final manuscript. DS and CR helped prepare the figure.

## Conflict of Interest Statement

The authors declare that the research was conducted in the absence of any commercial or financial relationships that could be construed as a potential conflict of interest.
